# Nonrecurrent 17p duplications in two patients with developmental and neurological abnormalities

**DOI:** 10.1038/s41439-025-00310-6

**Published:** 2025-03-26

**Authors:** Ah Jin Lee, Byung Kwon Pi, Soo Hyun Nam, Hyun Su Kim, Byung-Ok Choi, Ki Wha Chung

**Affiliations:** 1 Department of Biological Sciences, Kongju National University, Gongju, Republic of Korea; 2 Cell and Gene Therapy Institute, Samsung Medical Center, Seoul, Republic of Korea; 3 Department of Radiology, Samsung Medical Center, Sungkyunkwan University School of Medicine, Seoul, Republic of Korea; 4 Department of Neurology, Samsung Medical Center, Sungkyunkwan University School of Medicine, Seoul, Republic of Korea

**Keywords:** Disease genetics, Disease genetics

## Abstract

Variable copy number variations (CNVs) in the short arm of chromosome 17 are associated with many neurodevelopmental disorders, including Charcot–Marie–Tooth disease type 1A, Potocki–Lupski syndrome and Yuan–Harel–Lupski syndrome. Here we examined CNVs in two sporadic cases of developmental abnormalities, brain impairment and peripheral neuropathy. We identified novel duplications of approximately 14.1 Mb at 17p11.2–p13.1 (containing *PMP22* and *RAI1*) and 17.6 Mb at 17p11.2–p13.3 (*YWHAE*, *PAFAH1B* and *PMP22*) in each patient. Both duplications were suggested to be produced by de novo mutations of paternal origin. This study suggests that CNVs at 17p should be examined in patients with peripheral neuropathy as well as developmental and brain abnormalities.

The short arm of human chromosome (17p) is regarded as a region of genomic instability; thus, many copy number variations (CNVs) in this region have been reported to be associated with syndromes or diseases that manifest developmental and neurological abnormalities. Duplication and deletion of 1.4 Mb in 17p12, including *PMP22*, were first reported to be associated with peripheral neuropathies of Charcot–Marie–Tooth disease type 1A (CMT1A) and hereditary neuropathy with liability to pressure palsies (HNPP), respectively^1,2^. Potocki–Lupski syndrome (PTLS), characterized by hypotonia, mental and behavioral abnormality, is usually caused by a recurrent duplication of 17p11.2, including the *RAI1* gene^3,4^. Smith–Magenis syndrome (SMS), which reveals overlapping clinical features with PTLS, is caused by a reciprocal deletion of 17p11.2 in SMS^5,6^. Yuan–Harel–Lupski syndrome (YUHAL) is caused by a duplication of 17p11.2–17p12, including both regions of CMT1A (17p12) and PTLS (17p11.2). Clinical features of YUHAL reveal blended phenotypes of PTLS and CMT1A with a potential earlier onset of neuropathy^7,8^. The reciprocal deletions of p11.2–17p12 have shown severe features of intellectual disability, speech and gross motor delays, behavioral problems and ocular abnormalities^9^. Miller–Dieker lissencephaly syndrome (MDLS), which shows symptoms of microcephaly, intellectual impairment, autism, cardiac malformations, growth retardation, dysmorphic facial features and reduced life expectancy, is caused by a deletion of 17p13.3 including *YWHAE* and *PAFAH1B1* (refs. ^9–11^). The same region is duplicated in 17p13.3 duplication syndrome (17p13.3DS), which shows considerable overlapping phenotypes with MDLS^9,12,13^. Patients with a 17p13.3 deletion, including *YWHAE* but excluding *PAFAH1B1*, showed consistent phenotypes in MDLS^14^. CNVs shown in patients with CMT1A, HNPP, PTLS and SMS are commonly related with nonallelic homologous recombination (NAHR)-mediated recurrent rearrangements involving low-copy repeat (LCR) sequences, while CNVs of the most YUHAL and MDS cases are associated with nonrecurrent replication-based fork stalling and template switching/microhomology-mediated break-induced replication^15^.

Except for CMT1A and HNPP, few cases of chromosomal rearrangements within 17p have been reported in East Asian countries^16–18^. This study examined two patients with complex phenotypes of developmental abnormalities and central and peripheral neuropathies (Fig. 1A). Their clinical phenotypes are presented in Supplementary Table 1. This study was approved by the institutional review boards of Samsung Medical Center (2014-08-057-002) and Kongju National University (KNU-IRB-2018-62). Informed consent was obtained from all the participants.

**Fig. 1:**
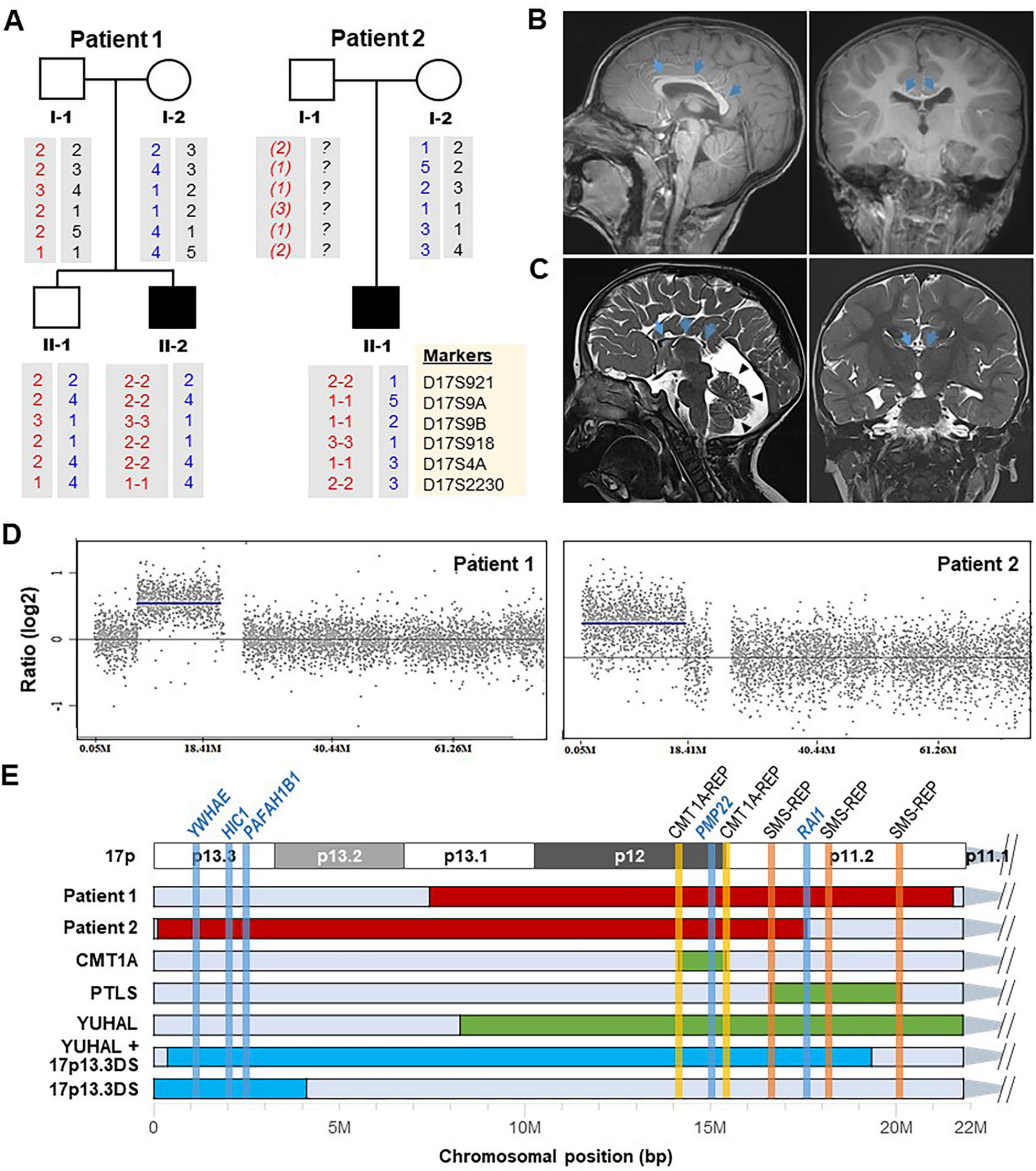
Two patients with duplications within 17p. **A** Pedigrees and haplotype analysis of microsatellites within 17p12. Paternal and maternal microsatellite haplotypes are indicated by red and blue, respectively. A putative haplotype shown in the father (I-1) in patient 2 is indicated by italics in parentheses. Repeat numbers of microsatellites are denoted by arbitrary numbers. **B**, **C** Brain MRI of patient 1 (**B** 2 years and 7 months) and patient 2 (**C** 2 years). Coronal and sagittal 3D T1- and T2-weighted spin echo images show diffuse thinning of the corpus callosum (arrows). Patient 1 shows a markedly decreased cerebellum volume within the posterior fossa (arrowheads). **D** Detection of duplications in 17p by aCGH. Gray dots and blue lines indicate aCGH probes and duplication regions, respectively. **E** Several cases with duplications in 17p. Duplication regions observed in this study (patients 1 and 2) are indicated by red, while common duplications causing CMT1A, PTLS and YUHAL are indicated by green. Previously reported several duplications are also shown by blue at the bottom. The vertical lines indicate LCRs (orange) and genes (blue). Duplication sizes and phenotypes of these cases are provided in Table 1.

Patient 1 (II-2 in Fig. 1A, left) was born with a low birth weight of 2.1 kg at 37 weeks of gestation. There was no family history. He was hospitalized for 2 weeks owing to aspiration pneumonia at 1 month of age. He could stand on his own at 12 months of age and walk on his own at 24 months of age. He had slow growth and development compared with his peers and a speech impediment. He walked unsteadily and could not run. His hand movements were clumsy, and he had difficulty dressing. A neurological examination at age 9 showed muscle weakness and sensory dysfunction in the upper and lower extremities. Muscle weakness was more severe in the distal than in the proximal part. Deep tendon reflex was decreased in the upper and lower extremities. Foot deformities were observed in both feet. Nerve conduction studies showed demyelinating sensorimotor polyneuropathy. Brain magnetic resonance imaging (MRI) obtained at the age of 2 years and 7 months revealed diffuse thinning of the corpus callosum with individual thickness measured at the genu, body, isthmus and splenium below the third percentile of the reported reference data of the similar age group (Fig. 1B).

Patient 2 (II-1 in Fig. 1A, right) was born with a low birth weight at 37 weeks of pregnancy. He showed developmental delay and hypotonia. Rehabilitation physical and speech therapy were started at 6 months of age. He could walk without assistance for the first time at the age of 3. Thereafter, he continued to walk unsteadily and was unable to run. He had difficulty dressing and was impaired in daily life. A neurological examination performed at age 4 showed decreased reflexes of the lower extremities and foot deformities in both feet. Cerebellar examination could not be performed due to lack of cooperation. An X-ray examination at age 4 showed a dislocation of the left hip joint. His speech was babbling, and he had difficulty expressing himself. Brain MRI showed a paper-thin appearance of the corpus callosum with individual thickness measured at the genu, body, isthmus and splenium below the third percentile of the reported reference data of the similar age group (Fig. 1C). Cerebellar atrophy was also noted. He also underwent an MRI of the lower extremities at the age of 4 years and 9 months. Fat-suppressed T2-weighted spin echo images showed diffuse enlargement with increased signal intensities of the fascicles of bilateral sciatic, tibial and peroneal nerves, findings consistent with a demyelinating neuropathy (Supplementary Fig. 1). Notable intramuscular fat infiltration suggesting muscle denervation was observed on T1-weighted spin echo images.

The 17p12 (*PMP22*) duplication was first identified in both patients by newborn genetic screening and confirmed by a multiplex microsatellite PCR and real-time PCR^19^. Because both cases showed complex phenotypes beyond CMT1A, we applied an array comparative genomic hybridization (aCGH) as a subsequent genetic analysis using the SurePrint G3 Human CGH 4x180k Microarray platform (Agilent). The aCGH revealed novel duplications of approximate sizes of 14.1 Mb (7.40–21.50 Mb) at 17p11.2–p13.1 in patient 1 and 17.6 Mb (0.06–17.63 Mb) at 17p11.2–p13.3 in patient 2 (Fig. 1D). Haplotypes of six microsatellites (D17S921, D17S9A, D17S9B, D17S918, D17S4A and D17S2230) within the 17p12 duplicated region were identical to one of two in the fathers in both patients: 2-2-3-2-2-1/2-2-3-2-2-1 in patient 1 and 2-1-1-3-1-2/2-1-1-3-1-2 in patient 2 (Supplementary Fig. 2). The duplication including the *PMP22* and *RAI1* genes in patient 1 was novel but similar to the duplications seen in some patients with YUHAL^8,15^. The relatively long novel duplication in patient 2 contained *YWHAE*, *PAFAH1B*, *PMP22* and upstream sequence (promoter and 5′-untranslated region (5′-UTR)) of *RAI1* (Supplementary Fig. 3). We supposed that *RAI1* did not contribute to the phenotypes observed in patient 2 because no coding axon was included in the duplication region.

The complex phenotypes of patient 1 are consistent with those of patients with YUHAL^5,8^. In patient 2, the clinical phenotypes are matched with previous cases of 17p13.3DS shown in Fig.  1E and Table 1^12,16^, which suggests that the genomic overdosage of *YWHAE* and *PAFAH1B* is majorly responsible for the phenotypes, as discussed by Curry et al.^12^. The additional phenotype of demyelinating sensorimotor polyneuropathy in patient 2 was probably due to the *PMP22* duplication.

Patient 1 was determined to have a de novo mutation originating from the unaffected father by the haplotype analysis of six microsatellites within the 17p12 region (Fig. 1A). No genetic testing was performed on the father of patient 2; however, a paternal-origin de novo mutation is suggested, as the mother passed a normal haplotype to the affected son (Fig. 1A). The identification of the de novo mutations is consistent with the frequent observation of de novo mutations in severe patients with CNVs within 17p in previous reports^8,12^. Both duplicated regions showed the same haplotypes with each original template, suggesting replication-based duplication rather than unequal crossing over between nonsister chromatids. However, because these duplications were determined only by the aCGH method, we cannot rule out the involvement of other types of duplication, such as unbalanced translocation, instead of the tandem duplication. Table 1 presents the variable duplications within the 17p and their phenotypes, including the present two cases.

**Table 1:** Patients with variable duplications within the 17p and their phenotypes.

Patients^a^ (types)	Map	Size (Mb)	Key genes	Clinical phenotypes	References
Patient 1 (YUHAL)	17p11.2–13.1	14.1	*PMP22*, *RAI1*	Developmental delay, thin corpus callosum, demyelinating peripheral neuropathy	This study
Patient 2 (CMT1A + 17p13.3DS)	17p11.2–13.3	17.6	*YWHAE*, *HIC1*, *PAFAH1B*, *PMP22*, *RAI1* (only promoter and 5′-UTR)	Developmental delay, thin corpus callosum, cerebellar atrophy, hypotonia	This study
CMT1A	17p12	14.0	*PMP22*	Demyelinating peripheral neuropathy	^1^
PTLS	17p11.2	3.7	*RAI1*	Hypotonia, short stature, mental retardation, behavioral abnormalities	^3^
YUHAL	17p11.2–13.1	13.4	*PMP22*, *RAI1*	Complex phenotypes of CMT1A and PTLS	^15^
YUHAL + 17p13.3DS	17p11.2–13.3	19.0	*YWHAE*, *HIC1*, *PAFAH1B*, *PMP22*, *RAI1*	Intrauterine growth retardation and single umbilical artery, agenesis of the corpus callosum, hearing loss, scoliosis, hypotonia, hydrocephalus, atrial septal defect, patent ductus arteriosus, seizure	^16^
17p13.3DS	17p13.3	4.1	*YWHAE*, *HIC1*, *PAFAH1B*	Ventriculomegaly (prenatal), round face, cerebellar vermis hypoplasia, hypotonia, intellectual disability	^12^

^a^All the patients (or disorders) are the same cases as those shown in Fig. 1E.

In conclusion, we identified two Korean patients with novel nonrecurrent duplications at 17p and confirmed the genotype–phenotype correlation. Because both patients were initially diagnosed as having CMT with some additional symptoms, this study suggests examination of CNVs at 17p for patients with peripheral neuropathy concurrent with developmental and brain abnormalities for exact diagnosis.

## HGV datbase

The relevant data from this Data Report are hosted at the Human Genome Variation Database at 10.6084/m9.figshare.hgv.3485 and 10.6084/m9.figshare.hgv.3488.

## Supplementary information

Supplementary Table 1

Supplementary Fig. 1

Supplementary Fig. 2

Supplementary Fig. 3
