# Supplementary material for: Nonrecurrent 17p duplications in two patients with developmental and neurological abnormalities
Source: Hum Genome Var. 2025 Mar 26;12:6. doi: 10.1038/s41439-025-00310-6 (PMC11947145; doi:10.1038/s41439-025-00310-6)
Supplement: Supplementary file 1 — Supplementary Table 1 [file 41439_2025_310_MOESM1_ESM.docx]

**Supplementary Table S1.** Clinical manifestations of patients with 17p rearrangements

| Patients | Patient 1 (II-2) | Patient 2 (II-1) |
| --- | --- | --- |
| Age at examination (years) | 9 | 4 |
| Age at onset (years) | < 1 | < 1 |
| Age at walking (years) | 2 | 3 |
| Weight (kg) / height (cm) / BMI (kg/m^2^) | 19.8 / 152 / 8.6 | 16.0 / 115 / 12.1 |
| Limb muscle weakness (MRC) |  |  |
| Upper proximal /distal | 4+ / 4+ | 4+ / 4+ |
| Lower proximal / distal | 4+ / 4+ | 4+ / 4+ |
| Muscle atrophy | Moderate | Moderate |
| Sensory disturbance | Yes | Yes |
| DTR |  |  |
| Biceps jerk reflex | Decreased | Decreased |
| Knee jerk reflex | Decreased+ | Decreased |
| Foot deformity | Yes | Yes |
| Developmental delay | Yes | Yes |
| Pyramidal sign | No | No |
| Dysarthria / Nystagmus | No / No | No / No |
| Intellectual disability | Yes | Yes |
| Hearing loss | No | No |
| Lower leg MRI | ND | Marked nerve hypertrophy |
| Brain MRI | Thin corpus callosum | Thin corpus callosum, cerebellar atrophy |
| Nerve conduction study^a^ |  |  |
| Median CMAP (mV) / MNCV (m/s) | 4.6 / 16.6 | ND |
| Peroneal CMAP (mV) / MNCV (m/s) | 4.4 / 15.9 | ND |
| Sural SNAP (μV) / SNCV (m/s) | 4.5 / 16.3 | ND |

Abbreviations: BMI: body mass index, CMAP: compound muscle action potential, DTR: deep tendon reflex, ND: not done, MNCV: motor nerve conduction velocity, MRC: medical research council scale, MRI: magnetic resonance imaging, SNAP: sensory nerve action potential, SNCV: sensory nerve conduction velocity.

^a^ Normal values: median MNCV ≥ 50.5 m/s, peroneal MNCV ≥ 41.2 m/s, sural SNCV ≥ 32.1 m/s, median CMAP ≥ 6 mV, peroneal CMAP ≥ 1.6 mV, and sural SNAP ≥ 6.0 μV.
