# Supplementary material for: Nonrecurrent 17p duplications in two patients with developmental and neurological abnormalities
Source: Hum Genome Var. 2025 Mar 26;12:6. doi: 10.1038/s41439-025-00310-6 (PMC11947145; doi:10.1038/s41439-025-00310-6)
Supplement: Supplementary file 2 — Supplementary Fig. 1 [file 41439_2025_310_MOESM2_ESM.docx]

**
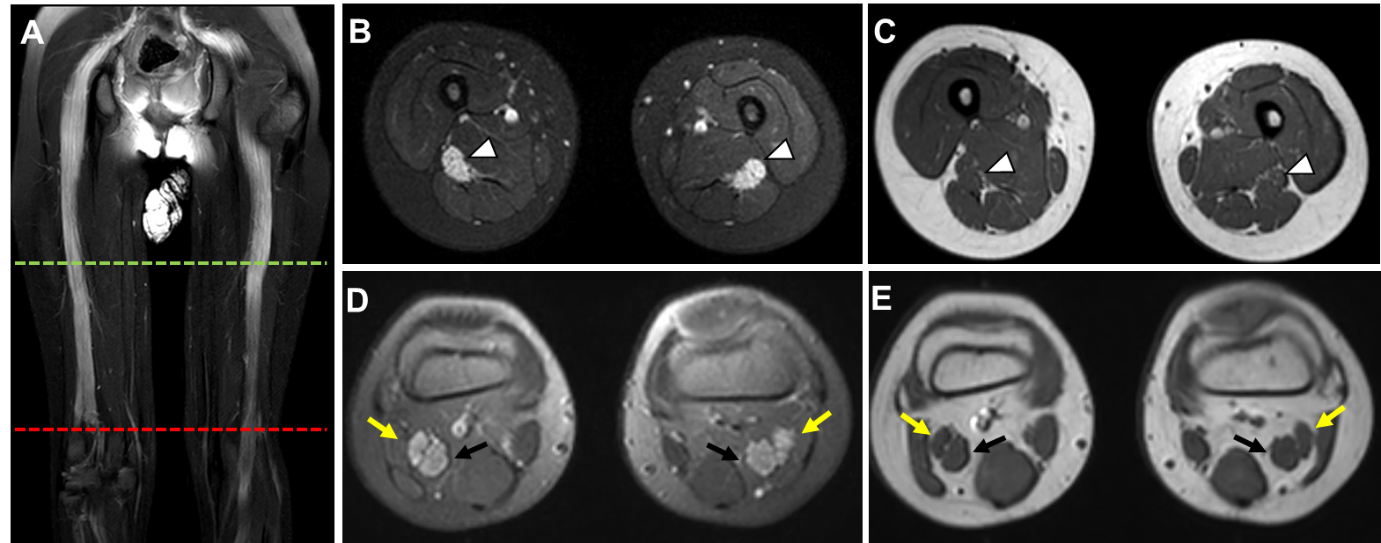
**

**Supplementary Figure S1.** Lower extremity MRIs at the age of 4 years and 9 months for patient 2. Coronal (A) and axial fat-suppressed T2-weighted spin echo images acquired at the proximal and distal thigh (B-E) showed diffuse enlargement with increased signal intensities of bilateral sciatic (arrowheads), tibial (black arrows), and peroneal nerve fascicles (yellow arrows)
