# Supplementary material for: Nonrecurrent 17p duplications in two patients with developmental and neurological abnormalities
Source: Hum Genome Var. 2025 Mar 26;12:6. doi: 10.1038/s41439-025-00310-6 (PMC11947145; doi:10.1038/s41439-025-00310-6)
Supplement: Supplementary file 3 — Supplementary Fig. 2 [file 41439_2025_310_MOESM3_ESM.docx]

**
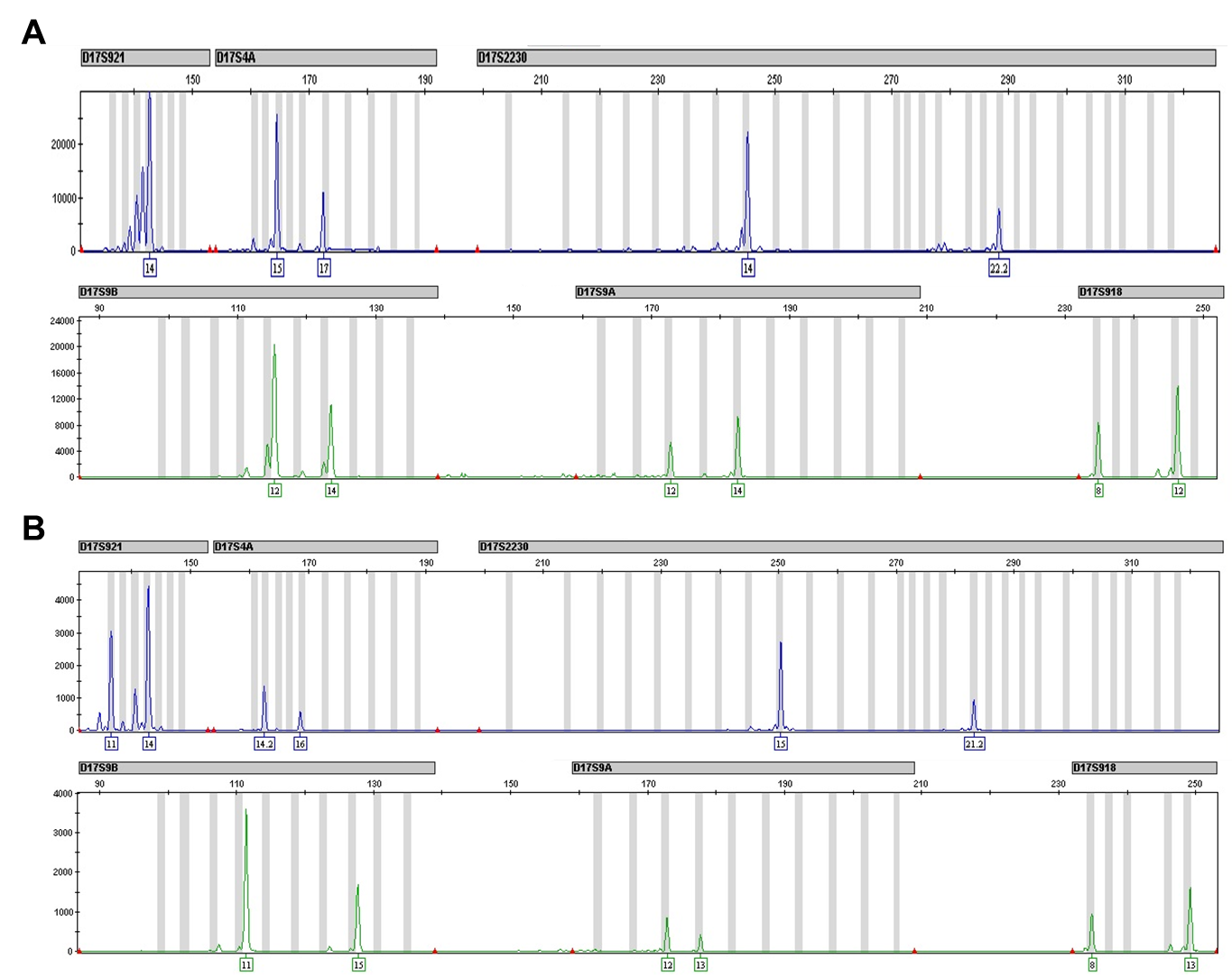
**

**Supplementary Figure S2.** Genotyping chromatograms of six microsatellites (D17S921, D17S9A, D17S9B, D17S918, D17S4A and D17S2230) within the 1.4 Mb CMT1A duplication region in chromosome 17p12. Six microsatellites were simultaneously amplified by a multiplex PCR method using FAM- or HEX-labeled primers (blue: FAM, green: HEX). The PCR products were resolved using the SeqStudio Genetic Analyzer (Life Technologies-Thermo Fisher Scientific, USA), and genotypes were determined using the Genotyper program (Applied Biosystems, USA). The DNA size ranges are shown at the top of the chromatograms (A: patient 1, B: patient 2)
