# Supplementary material for: Nonrecurrent 17p duplications in two patients with developmental and neurological abnormalities
Source: Hum Genome Var. 2025 Mar 26;12:6. doi: 10.1038/s41439-025-00310-6 (PMC11947145; doi:10.1038/s41439-025-00310-6)
Supplement: Supplementary file 4 — Supplementary Fig. 3 [file 41439_2025_310_MOESM4_ESM.docx]

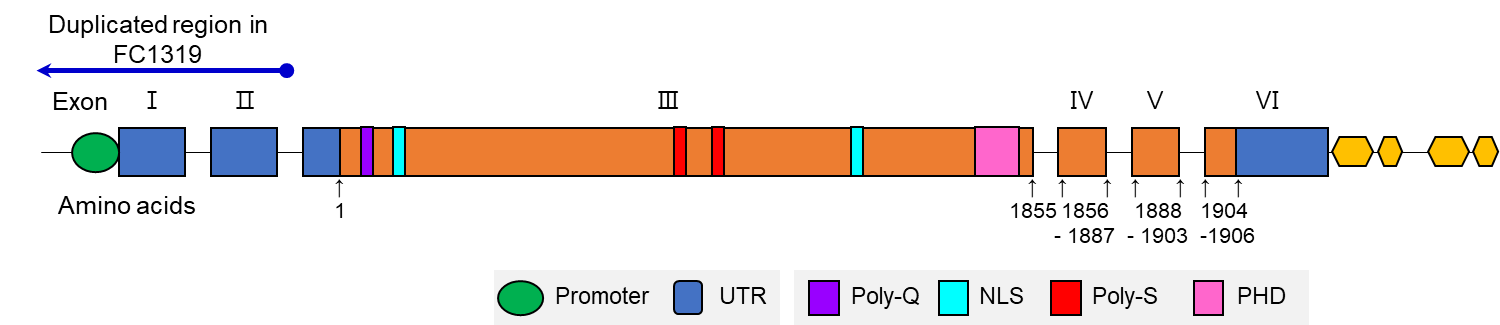


**Supplementary Figure S3.** Schematic diagram of *RAI1* gene which is a key gene associated with Potocki-Lupski syndrome and Smith-Magenis syndrome. Promoter, exons, and several protein domains are provided in the diagram. The duplication region shown the patient FC1319 (corresponding upstream elements including promoter and 5-UTR) is indicated at the top of diagram by a blue line (Poly-Q: glutamine-rich domain, NLS: bipartite nuclear localization signal, Poly-S: serine-rich domain, and PHD: C-terminal plant homeodomain).
